# Different Phases of Breast Cancer Cells: Raman Study of Immortalized, Transformed, and Invasive Cells

**DOI:** 10.3390/bios6040057

**Published:** 2016-11-28

**Authors:** Deepika Chaturvedi, Sai A. Balaji, Vinay Kumar Bn, Freek Ariese, Siva Umapathy, Annapoorni Rangarajan

**Affiliations:** 1 Department of Inorganic and Physical Chemistry, Indian Institute of Science, Bangalore 560012, India; deepika.5185@gmail.com (D.C.); vinaykbn07@gmail.com (V.K.B.); f.ariese@vu.nl (F.A.); 2 Molecular Reproduction, Development & Genetics, Indian Institute of Science, Bangalore 560012, India; balu.1pharma@gmail.com; 3 Department of Instrumentation and Applied Physics, Indian Institute of Science, Bangalore 560012, India; 4 Department of Pharmacology, Manipal College of Pharmaceutical sciences, Manipal 576104, India

**Keywords:** Raman spectroscopy, breast cancer, PC-LDA, LOOCV, lipid staining

## Abstract

Breast cancer is the most prevalent cause of cancer-associated death in women the world over, but if detected early it can be treated successfully. Therefore, it is important to diagnose this disease at an early stage and to understand the biochemical changes associated with cellular transformation and cancer progression. Deregulated lipid metabolism has been shown to contribute to cell transformation as well as cancer progression. In this study, we monitored the biomolecular changes associated with the transformation of a normal cell into an invasive cell associated with breast cancer using Raman microspectroscopy. We have utilized primary normal breast cells, and immortalized, transformed, non-invasive, and invasive breast cancer cells. The Raman spectra were acquired from all these cell lines under physiological conditions. The higher wavenumber (2800–3000 cm^−1^) and lower wavenumber (700–1800 cm^−1^) range of the Raman spectrum were analyzed and we observed increased lipid levels for invasive cells. The Raman spectral data were analyzed by principal component–linear discriminant analysis (PC-LDA), which resulted in the formation of distinct clusters for different cell types with a high degree of sensitivity. The subsequent testing of the PC-LDA analysis via the leave-one-out cross validation approach (LOOCV) yielded relatively high identification sensitivity. Additionally, the Raman spectroscopic results were confirmed through fluorescence staining tests with BODIPY and Nile Red biochemical assays. Furthermore, Raman maps from the above mentioned cells under fixed conditions were also acquired to visualize the distribution of biomolecules throughout the cell. The present study shows the suitability of Raman spectroscopy as a non-invasive, label-free, microspectroscopic technique, having the potential of probing changes in the biomolecular composition of living cells as well as fixed cells.

## 1. Introduction

Cancer is a complex disease in which a group of cells undergoes uncontrolled cell division. It is usually caused by an accumulation of a series of mutations that irreversibly transforms a normal cell into a cancerous one [1]. Cancer affects people of all nationalities; the risk of developing this disease increases with age [2]. The present study focuses on breast cancer, which is the most common form of cancer occurring in women worldwide. Most cases of breast cancer are diagnosed at anadvanced stage, as early diagnosis remains a challenge. Social barriers are one of the important factors that cause a delay in diagnosis of this disease. The increase in the number of patients with breast cancer is alarming [3]. Most of the cases (approx. 80%) of breast cancer are revealed when the woman feels a lump [4,5].

Currently, numerous techniques are being used in the field of medicine for early diagnosis and subsequent treatment. Despite tremendous advancement in technologies, it is still a challenge to diagnose cancer at its early stage, and this is a major cause of mortality. Techniques that are in common practice at the clinical level are ultrasound, X-ray imaging, magnetic resonance imaging (MRI), single photon emission computed tomography, positron emission tomography (PET), optical imaging, optical bioluminescence, fluorescence, etc. Most of the above mentioned techniques use X-rays or γ-rays, and are not without risk to the patient; another related problem is low resolution and sensitivity [6,7,8].

The limitations of these existing techniques warrant the quest for the development of a non-invasive, label-free, real-time, and highly sensitive method. One such promising non-invasive technique is Raman spectroscopy, which provides specific molecular signatures of a variety of materials, including biological samples [9,10,11]. Other important aspects of Raman spectroscopy are that it needs minimal sample preparation and is relatively insensitive to interference from water and hence easily applicable for in vivo studies of biological samples. Raman spectroscopy is a laser-based diagnostic tool that can provide unique biomolecular signatures of different types of cells, for instance in the vicinity of a tumor. It is based on the principle of inelastic scattering of photons by molecular vibrations. A small fraction of photons is scattered by interaction with vibrating chemical bonds, resulting in a shift towards lower and higher frequencies [12,13]. The energy differences between incident and scattered photons correspond to specific vibrational energies of the chemical bonds of the molecules. The Raman spectrum of a cell thus represents an intrinsic biochemical fingerprint and contains molecular-level information about the cellular components. This technique has been extensively applied to detect cancers in a wide range of organs including the cervix, skin, mouth, brain, prostate and ear [14,15,16,17,18,19,20,21,22]. Many studies have been published in the last decade using Raman spectroscopy as a non-invasive tool for the diagnostic analysis of breast cancer cells and tissues [23,24,25,26,27,28,29,30], where differences between cancerous samples and normal samples have been analyzed and explained. The present work has been undertaken to understand the progression of breast cancer by monitoring different phases of cancer systematically, which could suggest potential biomarkers of cancer progression and could be used for early detection. For that purpose, several cell lines have been chosen and analyzed, using Raman spectroscopy together with fluorescence imaging.

**Figure 1:**
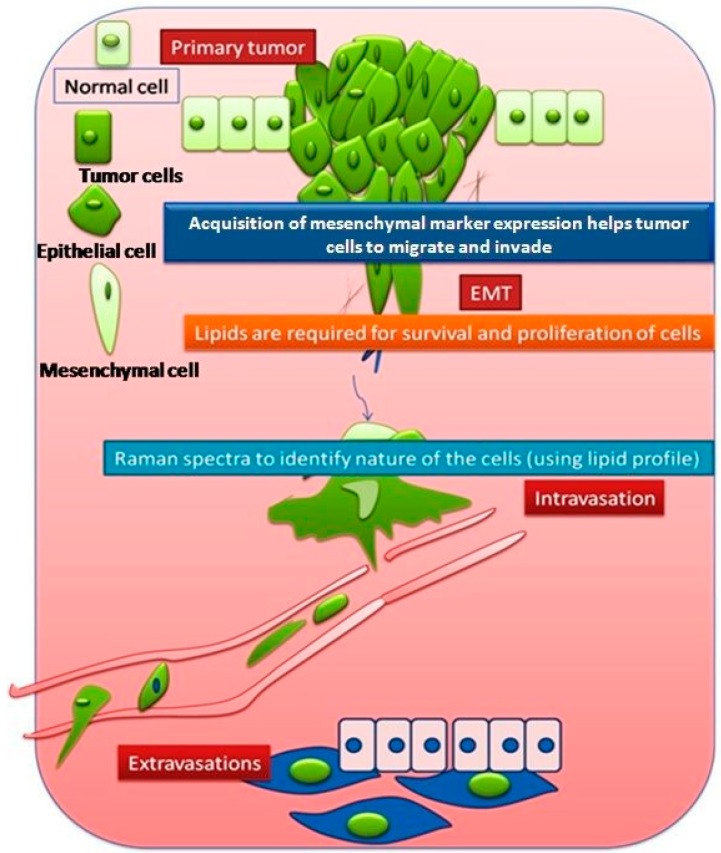
Graphical representation of primary and secondary tumor formation.

Epithelial cells are closely packed cells of the uppermost layer of skin or any tissue surface (upper lining of duct, etc.), whereas mesenchymal cells are supportive cells for the epithelial cells, loosely packed and surrounded by matrix. The process of conversion of normal cells into tumorigenic cells is termed cellular transformation. The detachment and subsequent invasion of cancer cells from the primary site to the secondary site involves a complex process known as epithelial–mesenchymal transition (EMT). In this process, epithelial cancer cells gain a more spindle-like morphology known as mesenchymal type, featuring enhanced migratory capacity, invasiveness, elevated resistance to apoptosis, and greatly increased production of extracellular matrix (ECM) components [31]. This favors the circulation and migration of the cancer cells from the primary site to a distant site where they may form a secondary tumor [32,33,34,35,36,37]. The EMT process is considered to be a prerequisite for cancer metastasis. Epithelial cells are considered to be terminally differentiated but activation of the EMT program changes the phenotype of the epithelial cells, resulting in the conversion of epithelial cells into mesenchymal cells. These processes are illustrated in Figure 1.

Invasion is the process of intravasation of cells from a primary tumor to the blood vessel and extravasation into the secondary site. EMTed cells play a major role in invasion in the process of metastasis.

Metastases are secondary tumors formed by cells that have been released from the initial or primary tumor and have reached other sites through blood vessels or lymphatics, or as a result of being shed into body cavities [38]. Metastasis can be portrayed as a two-phase process: the first phase involves the physical translocation of a cancer cell to a distant organ, whereas the second phase encompasses the ability of the cancer cell to develop into a metastatic lesion at that distant site [39]. Histological evidence largely suggests that metastatic tumors have the same kind of cells as the primary tumor. However, it is obvious that an invasive cell must have characteristics contributing to the process of metastasis that are different from non-metastatic cells. Detection of these specific characteristics could be of immense value for the clinical diagnosis and treatment of cancer. Our aim is to identify these characteristics using Raman spectroscopy.

To monitor the biochemical changes associated with the process of cellular transformation and cancer progression, seven different human cell lines were chosen including normal, immortalized, EMTed, noninvasive, and invasive carcinoma (HMECs, HMLE, HMLE-Twist, T47D, HMLE-Ras, BT-474, and MDAMB231).

Phase contrast images revealing the cellular morphology of all these cells are shown in Supplementary Figure S1. Among these cells, HMECs are primary normal breast tissue-derived cells, HMLE are immortalized non-cancerous breast cells [40], and HMLE-Twist comes in the category of immortalized and EMTed cells [41]. BT-474 and T47D are non-metastatic-non-invasive breast cancer epithelial cell lines, HMLE-Ras is an immortalized and transformed (tumorigenic) cell line, and MDAMB231 cell is a highly invasive/metastatic breast epithelial cancer cell line.

The main objectives of the present study were (i) to study the biochemical profiles of the various cell lines representing different stages of cancer and (ii) to differentiate between non-invasive and invasive cells based on their biomarkers, using Raman spectroscopy as a novel technique, together with biological validation using fluorescence staining. In this way, the present study could provide information on the biochemical changes associated with the conversion of normal cells into immortal and tumorigenic cells, as well as the differences between invasive and non-invasive cancer cells.

## 2. Materials and Methods

### 2.1. Selection of Cell Lines and Cell Culturing

HMECs were obtained as described previously [42] and cultured in DMEM-F12 media containing 10 ng/mL hEGF, 0.5 μg/mL hydrocortisone, and 10 μg/mL insulin (Sigma-Aldrich, St. Louis, MO, USA), heparin 20 ng/mL and B27 from Invitrogen (Carlsbad, CA, USA) along with penicillin (1 KU/mL), streptomycin (0.1 mg/mL), gentamycin (8 mg/mL), and Fungizone (0.5 μg/mL). HMLE and HMLE-Ras [40] were cultured in DMEM-F12 media containing 10 ng/mL hEGF, 0.5 μg/mL hydrocortisone, and 10 μg/mL insulin (Sigma-Aldrich), along with penicillin (1 KU/mL), streptomycin (0.1 mg/mL), and Fungizone (0.5 μg/mL).

HMLE cells overexpressing Twist (HMLE-Twist) were generated by retroviral transduction [41]. T47D and BT-474 were cultured in RPMI (Sigma-Aldrich) supplemented with 10% Fetal Bovine Serum (FBS), penicillin (1 KU/mL), and streptomycin (0.1 mg/mL) (Invitrogen). Breast epithelial cell lines MDAMB231 were cultured in Dulbecco’s Modified Eagle’s Medium (DMEM) (Sigma-Aldrich) supplemented with 10% Fetal Bovine Serum (FBS), penicillin (1 KU/mL), and streptomycin (0.1 mg/mL).

The cells were cultured at an optimum temperature of 37 °C with 5% CO_2_ atmosphere in a sterile incubator. Once the cells reached approximately 90% confluence in the culture dish, the medium was removed and cells were rinsed with 1x PBS buffer to remove remnants of medium. Later, the cells were trypsinized using 0.25% trypsin (from Invitrogen) in 0.53 mM EDTA to disperse the cell layer and obtain single cells. DMEM+10% FBS media were then added to quench the action of trypsin. Trypsinized cells were then centrifuged at 1200 rpm for 3 min at room temperature. The pellet obtained after centrifugation was re-suspended in fresh growth medium. The suspension containing single cells was then plated onto MgF_2_ cover slips (Global Optics (UK) Ltd., Bournemouth, UK) containing the original growth medium and incubated in the incubator.

### 2.2. For Live Cell Experiments

MgF_2_ cover slips were thoroughly rinsed with 1x PBS buffer prior to Raman experimental analysis to wash off medium remnants. Spectra were acquired from each of these cell lines adhered to different MgF_2_ coverslips and suspended in PBS buffer to maintain the optimum conditions required for the viability of cells. Each of the spectra was taken from a different point in the cytoplasm of the cell. Three to four Raman spectra were acquired from each cell and for each experiment seven to eight cells were considered for collecting Raman spectra, resulting in 30 spectra per cell type.

### 2.3. Preparation of Cells for Fixed Cell Experiments

For Raman image analysis, cells were cultured on MgF_2_ coverslips. Samples were maintained in growth medium until the time of the Raman experiment. The growth medium was aspirated from all samples prior to fixation and they were washed several times with PBS. Then the washed slides were dipped in methanol/acetone (1:1 ratio) and kept at −20 °C for 5 min. Methanol and acetone were procured from Merck (Kenilworth, NJ, USA). After that, the slides were kept outside for drying at room temperature.

### 2.4. Raman Microspectroscopy

#### 2.4.1. Raman Spectroscopy of Live Cells

Raman spectra of all the seven cell lines were measured using a Renishaw InVia Raman spectrometer connected to a Leica microscope (Leica Microsystems CMS GmbH, Wetzlar, Germany). A 785 nm diode line focus laser (~28 mW at the sample) was focused on the cells with the help of a 50XL (NA = 0.50) long working distance objective; the laser spot size was ~6 μm^2^. The laser power on the sample was optimized so as to avoid any damage to the cells. A Peltier cooled charge coupled device (CCD) detector (1024 × 256 pixels) fitted to a 1200 grooves/mm grating spectrometer was used to detect the signal. Spectra were collected at 25 s acquisitions, once for the lower wavenumber (LWN) window (700–1800 cm^−1^) and once for the higher wavenumber (HWN) window (2800–3000 cm^−1^). Cells of similar size and shape were selected and care was taken to ensure that each spectrum was collected from a new location in the cell. A standard cell viability assay was performed on the cells after the Raman experiments using Trypan Blue dye [43] (Sigma) and shown in Figure S2. The instrumental calibration was verified using the silicon line at 520 cm^−1^. The observed data were processed and analyzed using Origin 8.5 software (Origin Lab Corporation, Northampton, MA, USA). The acquired Raman spectra matched well with those on human cell lines published in the literature [44,45,46].

#### 2.4.2. Raman Imaging of Fixed Cells

Raman images of a fixed cell from each of the seven cell lines were acquired in the HWN window to obtain the spatial distribution of biomolecules within the cells. Isolated single cells were raster-scanned with 1 μm step size through the same objective (50XL). Raman backscattered light from each point of the cell was accumulated over 15 s. The typical measurement time for the Raman map was ~3 h. Because of the long mapping times required to obtain a sufficient signal-to-noise ratio, Raman maps were acquired for only a single cell per cell line, and only at one spectral range (HWN). Power at the sample was ~28 mW.

#### 2.4.3. Data Analysis

Spectral artifacts (e.g., baseline fluctuations, differences in thickness or water content, noise, and cosmic rays) can have a considerable effect on the interpretation of data, so it is important to distinguish between biochemical information and undesired effects. For these reasons, pre-processing of the spectral data should be carried out [47,48]. For pre-processing analysis, Origin version-8.5 software was used. Ten-point baseline correction for the LWN range and three-point base line correction for the HWN range were applied to each of the spectra. For reducing the noise, spectra were smoothed using a Savitsky–Golay filter (five points, second-order polynomial). All spectra were normalized by dividing each point by the norm of the whole spectrum. The contribution from the PBS + MgF_2_ coverslip, which adds to the background in the spectra, was acquired at the same time and is given in the Supplementary Materials (Figure S3). Corresponding peaks (990 cm^−1^ and 1640 cm^−1^) are marked with an asterisk in the experimental results. The 990 cm^−1^ peak is due to phosphate; because of its narrow spectral width, it does not significantly interfere with the neighboring phenylalanine peak at 1002 cm^−1^ (verified with curve fitting analysis, not shown). The background peak present at 1640 cm^−1^ is due to the presence of water and shows a strong overlap with the Amide I Raman band at 1654 cm^−1^ in the live cell Raman spectra [48]. This background will not be identical inside and outside the cell and may change due to evaporation, so a background subtraction could potentially result in artifacts. Therefore, in the present work we have not considered the Amide I Raman band for quantitative analysis.

*Multivariate Analysis:* Furthermore, we have performed multivariate analysis for the three groups of cell lines, using the preprocessed spectral data. We have utilized Principal Component–Linear Discriminant Analysis (PC-LDA). PC-LDA is a method that employs PCA based on a set of principal components to best describe the within-group variance, and LDA to maximize the variance between different groups using the principal components as input. In principle, PCA reduces the dimension of the data based on the principal components (PCs) that describe the maximum variance in the spectral data (e.g., PC1, PC2, PC3, and so on). In the present analysis, the first three PCs were used. These PCs were subsequently used as inputs for performing LDA. We have used ~25 spectra per cell line for generating the PC-LDA model, and the performance of the model was tested using a leave-one-out cross-validation (LOOCV) approach.

### 2.5. Lipid Staining

Nile-Red and BODIPY (Invitrogen) staining was performed to measure the lipid levels in various breast cell lines. For lipid staining, 1 × 10^5^ cells were seeded in a 35 mm dish (glass bottom) and, after 24 h of seeding, Nile Red (1 µg/mL) was added and incubated in an incubator for 30 min. After incubation, cells were washed with 1X PBS and observed under a confocal microscope. Nile Red stains the hydrophilic lipids and is observed using the red color channel (excitation, 515–560 nm; emission, greater than 590 nm), whereas hydrophobic lipids like cholesterol esters and triglycerides are observed in the green color channel (excitation, 450–500 nm; emission, greater than 528 nm).

For BODIPY staining, after 24 h of seeding, the BODIPY reagent was added and incubated in the incubator for 30 min. After incubation, cells were washed with 1X PBS and observed under the confocal microscope (497 nm excitation and 503 nm emission). Image-Pro and GraphPad prism software were used to quantify the images and analyze the data. *p* values <0.05 were considered to be statistically significant. Statistical analysis was done using paired Student’s *t* test; *** represents *p* < 0.001, ** represents *p* < 0.01, and * represents *p* < 0.05.

## 3. Results and Discussion

### 3.1. Comparison between Primary (Normal), Immortalized, and Transformed Cells (in Live Conditions)

**Figure 2:**
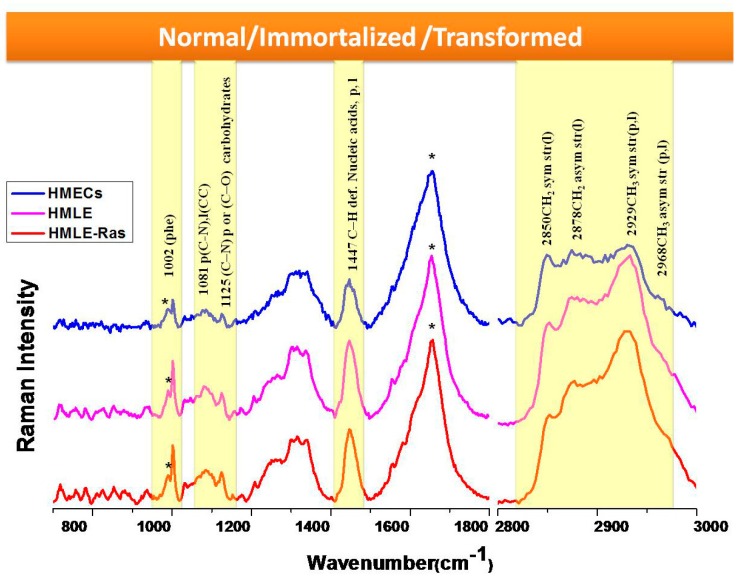
Raman spectra (700–1800 cm^−1^ and 2800–3000 cm^−1^) from live HMECs, HMLE, and HMLE-Ras cells. Each spectrum is an average of 30 spectra. Raman spectra were acquired in 25 s and had the baseline subtracted manually. Spectra are normalized and vertically offset for clarity. * = background, ** = water background + Amide I, p = protein, l = lipid.

Firstly, we compared three cell lines: HMECs as primary (normal) breast epithelial cells, HMLE as immortalized breast epithelial cells, and HMLE-Ras as transformed breast epithelial cells. This illustrated the transformation of normal cells to immortalized and transformed cells. For complete monitoring of this process, Raman spectra were acquired over both the LWN and the HWN range (Figure 2). The LWN (700–1800 cm^−1^) is known as the fingerprint region, which contains complete information about the biomolecules such as DNA, lipids, protein, nucleic acids, etc. The HWN (2800–3000 cm^−1^) is mostly used to establish the lipid profile of cells. We assigned all the prominent bands based on the published literature [44,45,46], as listed in Table 1. We observed prominent changes in the bands at 1447 cm^−1^ and 1002 cm^−1^. The Raman band centered at 1447 cm^−1^ corresponds to C–H deformation present in nucleic acids, proteins, and lipids. The Raman band observed at 1002 cm^−1^ is a marker peak for phenylalanine (ring breathing mode). Furthermore, we observed a change in ratio of the Raman peaks at 1081 cm^−1^ and 1125 cm^−1^. The Raman band centered at 1081 cm^−1^ has a contribution from C–N stretching modes in proteins and from C–C stretching modes in lipids. The other Raman band at position 1125 cm^−1^ has contributions from C–N stretching present in proteins and C–O present in carbohydrates. Therefore, the ratio of these two bands represents the relative lipid/carbohydrate levels. HMLE-Ras and HMLE cells show a relatively high intensity of the Raman band at 1447 cm^−1^ as observed from the results depicted in Figure 2. This could be due to a higher expression of nucleic acids, proteins, or lipids. The Raman band observed at 1002 cm^−1^ (phenylalanine) shows a comparatively lower intensity in the case of normal cells (HMECs). Furthermore, we found that the Raman band for tryptophan at 876 cm^−1^ and the skeletal mode of polysaccharides at 936 cm^−1^ also show a lower intensity in the case of normal cells. The lipid/carbohydrate ratio (1081/1125) is found to be higher in the case of immortalized cells (HMLE). Similarly, the Raman bands present in the HWN region show a relatively high intensity at 2929 cm^−1^ for transformed cells (HMLE-Ras), while the other Raman peak at 2878 cm^−1^ has a similar profile for both HMLE-Ras (transformed) and HMLE (immortalized) cells. The Raman band at 2850 cm^−1^ shows the highest relative Raman intensity change in the case of immortalized cells (HMLE). The Raman bands at 2850 and 2878 cm^−1^ were assigned to symmetric and asymmetric stretch vibrations of the many CH_2_ groups present in lipids. However, the peak at 2929 cm^−1^ is mainly a CH stretch mode of CH_3_ groups, which can be present in both lipids and protein, although CH_2_ groups in disordered chains may also have some contribution in this region [49]. Hence the results depicted in Figure 2 clearly indicate that transformed (HMLE-Ras) cells as well as immortalized cell lines show much higher lipid content than normal (HMECs) cells. On the other hand, a comparison of the transformed and immortalized cells shows that they both have similar lipid content. On closer inspection, we observe that immortalized cells show higher relative intensities of the Raman bands corresponding to the symmetric stretch of CH_2_ group and transformed cells show higher relative intensities of CH stretch Raman bands corresponding to the CH_3_ groups of both lipids and proteins.

### 3.2. Comparison between Non-Invasive and Invasive Cells (in Live Conditions)

**Figure 3:**
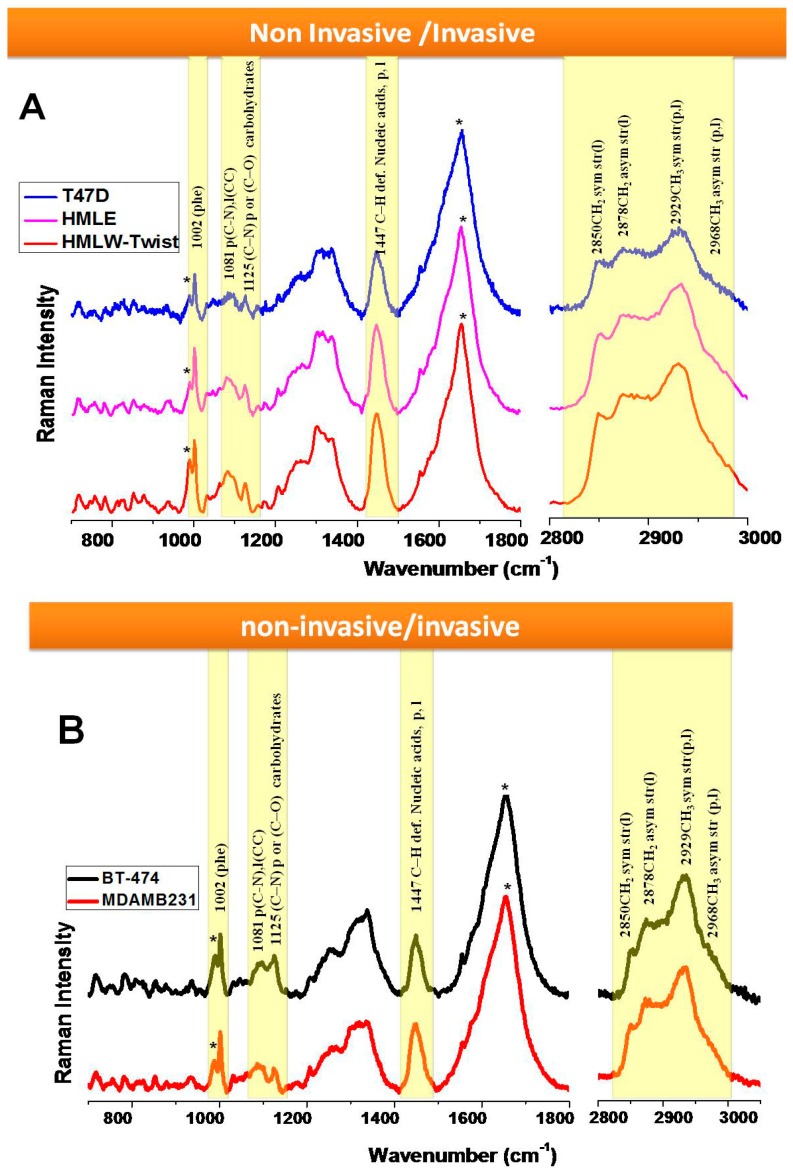
Raman spectra (700–1800 cm^−1^ and 2800–3000 cm^−1^) (**A**) from live T47D, HMLE, and HMLE-Twist cells; (**B**) from live BT-474 and MDAMB231 cells. Each spectrum is an average of 30 spectra. Raman spectra were acquired in 25 s and had the baseline subtracted manually. Spectra are normalized and vertically offset for clarity. * = background, ** = water background + Amide I, p = protein, l = lipid.

In the next part of our study, we compared the highly invasive breast epithelial cells (MDAMB231, HMLE-Twist) with non-invasive epithelial cells (BT-474, T47D, HMLE) as shown in Figure 3A,B. Interestingly, we observed that among these cell lines, the same bands show differences, as discussed in the previous section.

**Figure 4:**
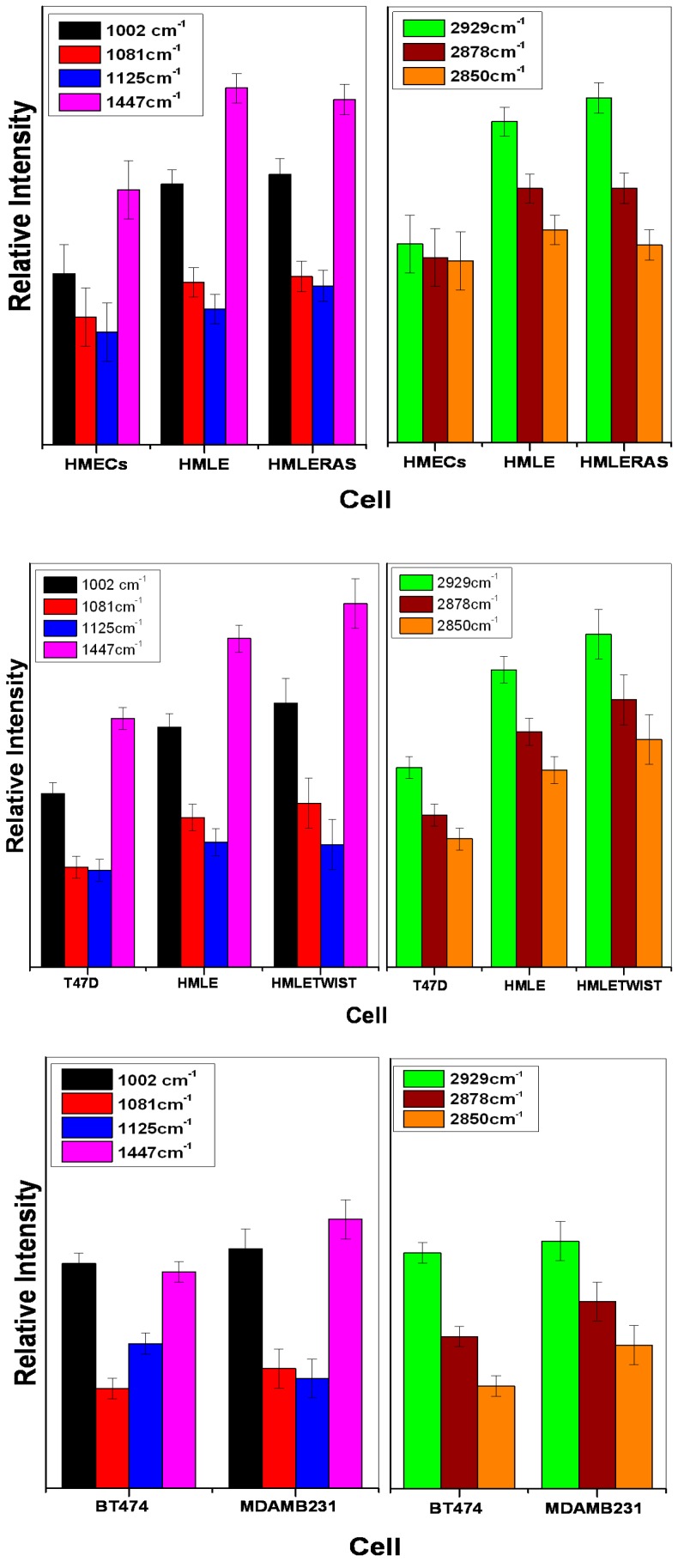
Illustration of Raman spectral intensity variation among the cell lines for specific peaks; left frames: LWN range: 1002 cm^−1^, 1081 cm^−1^, 1125 cm^−1^ and 1447 cm^−1^; right frames, HWN range: 2929 cm^−^^1^, 2878 cm^−1^, 2850 cm^−1^. Error bars show the range of lowest to highest intensity value (*n* = 30) after normalization (dividing each point by the norm of the whole spectrum).

White light images of MDAMB231 and HMLE-Twist, which are invasive cells, show that the shapes of these cells are spindle-like, while non-invasive cells show a characteristic patchy, epithelial circular morphology (Figure S1). We observed that the bands at 1447 cm^−1^ and 1002 cm^−1^ show differences in the Raman spectral profiles of the non-invasive and invasive cells as seen in Figure 3A, B. These two bands showed a higher intensity in the spectrum of HMLE-Twist (Figure 3A) and MDAMB231 (Figure 3B) in comparison with the non-invasive cells (Figure 4).

The lipid/carbohydrate ratio at 1081/1125 shows a higher value in the case of HMLE-Twist compared to T47D and HMLE (Figure 3A) and MDAMB231 compared to BT-474 (Figure 3B), which clearly indicates that the invasive cells have a higher lipid/carbohydrate ratio. We have also acquired the HWN region to see the lipid profile as it shows the CH stretching bands from lipids and proteins.

In the first comparison (Figure 3A), we observed that the T47D cell lines showed a much lower intensity of the lipid bands, as seen not only in the lower wavenumber (1447 cm^−1^) range but also in the higher wavenumber range. The other cell line that we considered non-invasive, i.e. HMLE, shows relatively intense Raman bands compared to T47D cells. This could be due to the fact that HMLE cells, although non-invasive in nature, are also known to be immortalized breast epithelial cells. We observed that among all three cell lines studied, the HMLE-Twist cells, which are the invasive form, showed the highest Raman intensity changes for lipid bands and also for the phenylalanine Raman band. The Raman spectral intensity variations within each cell line are given in Figure 4.

**Figure 5:**
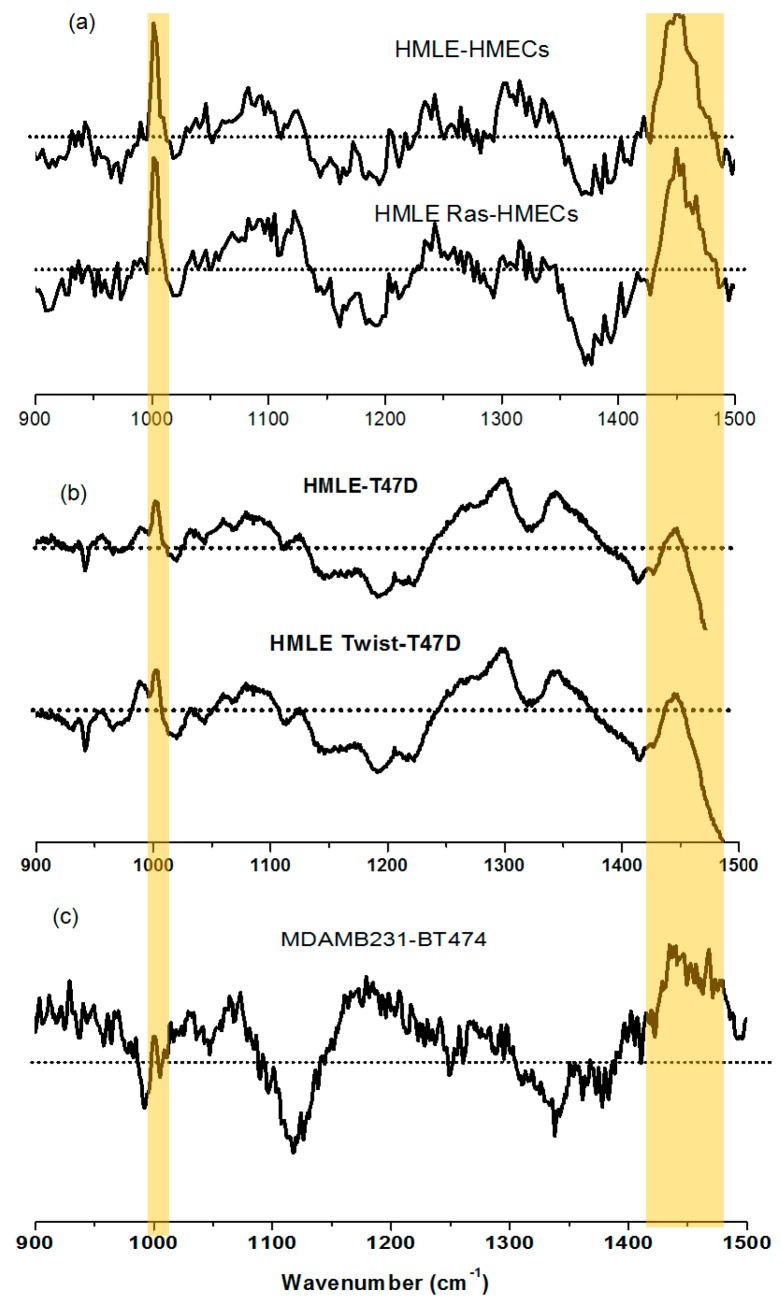
Difference Raman spectra for lower wavenumber range (**a**) (HMLE-HMECs) and (HMLE Ras-HMECs) cells (related to Figure 2) (**b**) (HMLE-T47D) and (HMLE Twist-T47D) cells (related to Figure 3A); and (**c**) (MDAMB231-BT474) cells (related to Figure 3B). The highlighted peaks indicate an increase of the Raman intensity at 1002 cm^−1^ (phenylalanine) and 1447 cm^−1^ (mostly lipids) in comparison with normal cells (HMECs), or least invasive cells (T47D and BT474).

**Figure 6:**
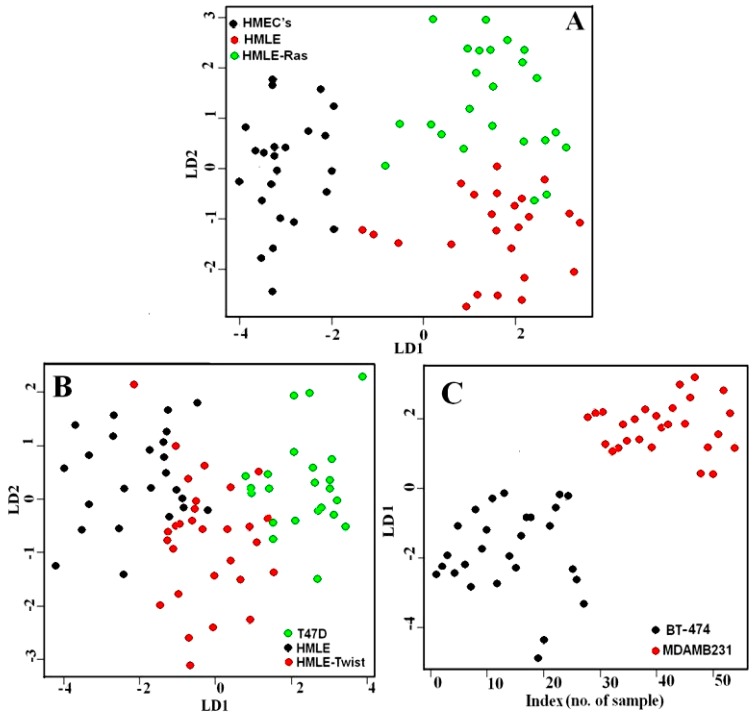
PC-LDA scores plot of (**A**) HMECs, HMLE and HMLE-Ras cells; (**B**) T47D, HMLE and HMLE-Twist cells; (**C**) BT-474 and MDAMB231 cells.

Next we compared BT-474 and MDAMB231 cell lines as non-invasive vs. invasive (see Figure 3B). We observed the 1447 cm^−1^ band and lipid /carbohydrate ratio to be the marker for the invasiveness of cells. We could also observe significant differences in the HWN range. The MDAMB231 cell line shows a higher Raman intensity for the 2850 and 2878 cm^−1^ bands compared to BT-474. Raman spectral signatures could differentiate between different groups such as (i) normal, immortalized, and transformed cells; or (ii) non-invasive and invasive cells. We also observed differences in other Raman bands that have not been highlighted in the manuscript. We focused on the Raman bands that showed very prominent and consistent changes and also have a connection with the available research on breast cancer. For more clarity, we have also plotted the difference spectra between the cell lines and these are given as Figure 5. For further evaluation of the capability of Raman spectra to differentiate between the cells associated with different phases of breast cancer, we performed Principal Component–Linear Discriminant Analysis (PC-LDA). From the classification results of PC-LDA, we observed from score plots that the data from all the cell lines clustered into distinct groups with a high degree of specificity and sensitivity (see Figure 6 and Table S1). The corresponding loading plots are shown in Supplementary Figure S4. The variability of the Raman spectra from highest to lowest intensity for the lower wavenumber range for all the cell lines is shown in Figure S5.

The PC-LDA classification results showed that the first set of spectral data for HMECs, HMLE, and HMLE-Ras cell lines separated with an accuracy of 83%. The second set of spectral data from T47D, HMLE and HMLE-Twist cell lines separated with 76% accuracy. The third set of spectral data from BT-474 and MDAMB231 cell lines were separated with 100% accuracy. The PC-LDA classification results are given in Table S1.

Comparison between primary (normal), immortalized, and transformed cells (Raman spectra of Figure 2 and Table S1) shows that all three cell lines, HMECs, HMLE and HMLE-Ras, form three separate clusters with a high specificity of 91%, 81%, and 93% respectively. Comparison between non-invasive and invasive cells T47D, HMLE and HMLE-Twist (Raman data of Figure 3A) shows separate clusters but we could also observe some overlap, which could be due to the fact that HMLE and HMLE-Twist are similar (immortalized vs. immortalized EMTed cells). The third plot, where we observed 100% specificity and sensitivity in classification, demonstrates the substantial spectral differences between epithelial cells BT474 and mesenchymal MDAMB231 cells (Raman spectra of Figure 3B and also Table S1).

The performance of the PC-LDA model was then validated using a leave-one-out cross-validation approach (LOOCV). The results are listed in Table S2, which shows that the PC-LDA model correctly identified the cells belonging to HMECs, HMLE and HMLE-Ras groups with an accuracy of approximately 92%. The cells from the T47D, HMLE, and HMLE-Twist groups were correctly identified with an accuracy of approximately 86%. Similarly, the BT474 and MDAMB231 cells were correctly identified with 100% accuracy. These results highlight the potential of the Raman spectroscopic approach combined with PC-LDA model analysis for the successful identification of cells corresponding to different phases of breast cancer.

### 3.3. Lipid Staining Assays

To validate the lipid profiles observed in the Raman spectra, standard lipid staining methods were undertaken. Mammalian cells can store excess lipid in cytosolic lipid droplets for several functions, such as storage of metabolic energy and formation of membrane components. Furthermore, lipids are also involved in protein storage and defense against viral replication [50,51].

**Figure 7:**
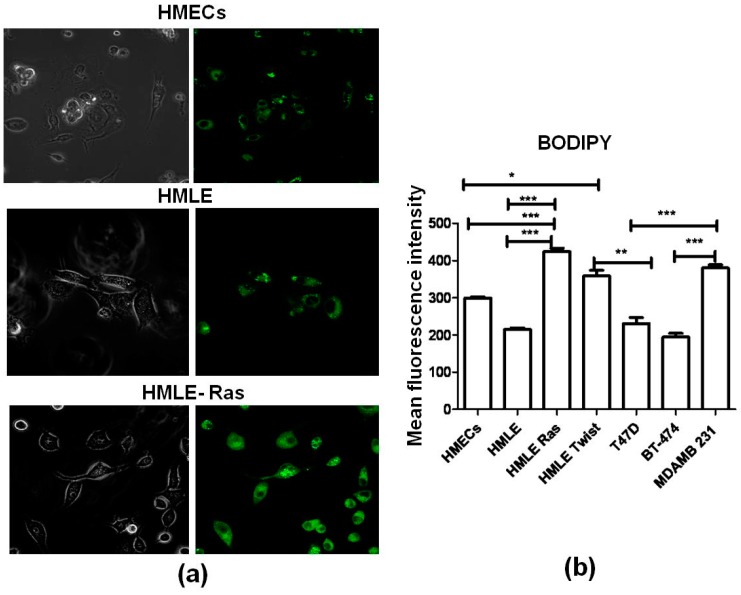
(**a**) Analysis of the phospholipid content in different cell lines (HMECs, HMLE, HMLE-Ras) using BODIPY staining. The left-hand images were observed in bright field; theright-handimages ware observed in green-fluorescence mode; 60× magnification in confocal microscope Olympus FV-10I). (**b**) Average fluorescence intensities (BODIPY 493/503) across different cell lines. Error bars represent standard error of the mean (SEM); (*n* = 3), where *n* is the number of experiments and significant differences are indicated by *.

**Figure 8:**
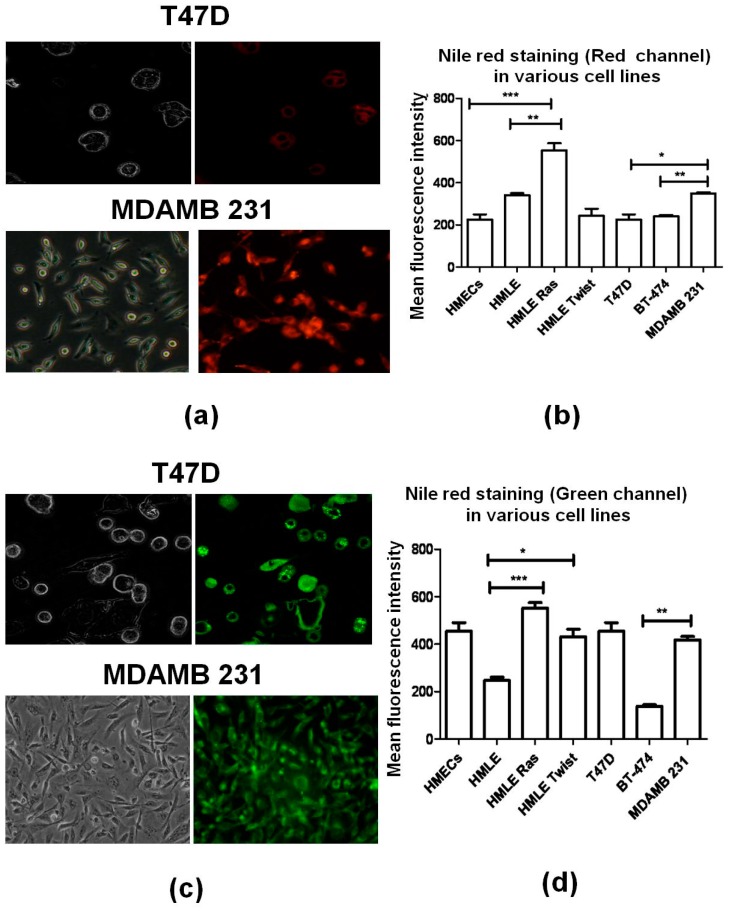
Analysis of the (frames **a**,**b**) hydrophilic fatty acid, and (frames **c**,**d**) hydrophobic fatty acid content in the cell lines T47D and MDAMB 231 using Nile Red staining. The left-hand images were observed in bright field; the right-hand images were observed in (**a**) red-fluorescence mode; and (**c**) green-fluorescence mode (60× magnification in confocal microscope-Olympus FV-10I); (**b**,**d**) Corresponding average Nile Red fluorescence intensities across different cell lines (**b**) red channel; excitation, 515–560 nm; emission, longer than 590 nm; (**d**) green channel, excitation, 450–500 nm; emission, longer than 528 nm). Error bars represent standard error of the mean (SEM); (*n*=3), where *n* is the number of experiments and significant differences are indicated by *.

Lipid rafts are membrane platforms for signaling molecules that regulate various cellular functions, including cell survival [52]. It is well established that lipid levels can be measured by two standard staining methods, i.e., BODIPY [53] (Figure 7) and Nile Red staining [54] (Figure 8). In the case of Nile Red staining we monitored two different channels, red and green. The red channel shows the presence of hydrophilic lipids (e.g., phospholipids), whereas the green channel represents hydrophobic lipids (e.g., cholesterol esters and triglycerides) present in cells.

From our Raman spectral data, we found that the lipid profile changes between different cell lines. In agreement with that, we observed that the transformed cells (HMLE-Ras cells) showed the highest fluorescence intensity in the case of BODIPY and Nile Red staining compared to normal (HMECs) and immortalized cells (HMLE). Similarly, from Figure 7 we conclude that the intensity of BODIPY fluorescence was also significantly higher in invasive cell lines (HMLE-Twist and MDAMB 231) compared to non-invasive cell lines (HMECs, HMLE, T47D, and BT-474). Interestingly, we observed that hydrophilic lipid levels were significantly higher in HMLE-Ras when compared with HMECs and HMLE. On the other hand, when we compare the hydrophobic lipid intensity of HMLE-Twist with HMLE and T47D we observed a higher intensity in the case of HMLE-Twist compared to HMLE. However, HMLE-Twist and T47D showed a similar intensity of hydrophobic lipids (Figure 8d). Furthermore, these three cell lines (T47D, HMLE, and HMLE-Twist) showed similar intensities of hydrophilic lipids (Figure 8b).

Finally, a comparison of non-invasive and invasive cells, i.e., BT-474 and MDAMB231, shows a higher intensity of hydrophobic lipids in the case of MDAMB231. However, we did not observe a significant Nile Red intensity difference with respect to hydrophilic lipids between MDAMB 231 and BT-474. On the other hand, invasive and transformed cells showed a significantly higher hydrophobic lipid content than non-invasive and non-transformed cells.

*Comparison of spectral intensity with lipid content (BODIPY and Nile-Red):* We compared the intensity of the Raman bands located at position 1447 cm^−1^ and 2929 cm^−1^ with the intensity of BODIPY staining and also with Nile Red staining (Figure S6). Within each group of cell lines, we have normalized the intensities to that of the control group (least invasive cell line). We observe in the left intensity plots that HMECs and HMLE-Ras show a similar trend, i.e., an increase in phospholipid content. However, in comparison to HMLE-Ras, HMLE shows a slight increase in Raman intensity, in contrast with the BODIPY staining intensity. Closer inspection shows that the hydrophilic lipid intensity has a similar trend as the Raman intensity: compared to normal cells (HMECs), immortalized (HMLE) and transformed cells (HMLE-Ras) have a higher lipid content.

In the next set (middle graphs) we compared T47D with HMLE and HMLE-Twist, which clearly demonstrates based on the Raman intensity that HMLE has more hydrophilic lipids and HMLE-Twist has an overall higher phospholipid content. Comparison of epithelial and mesenchymal cells (right graphs of Figure S6, BT474 and MDAMB231) shows a good matching of the Raman intensity with the staining intensity trends and shows a high overall lipid content in the case of mesenchymal MDAMB231 cells.

### 3.4. Raman Imaging of Fixed Cells

To visualize the spatial distribution of lipids, we have acquired Raman images from fixed cells as it is difficult to keep the cells alive for more than 3 h and maintain the same focus. We have mapped all cell lines over the HWN window (2800–3100 cm^−1^).

**Figure 9:**
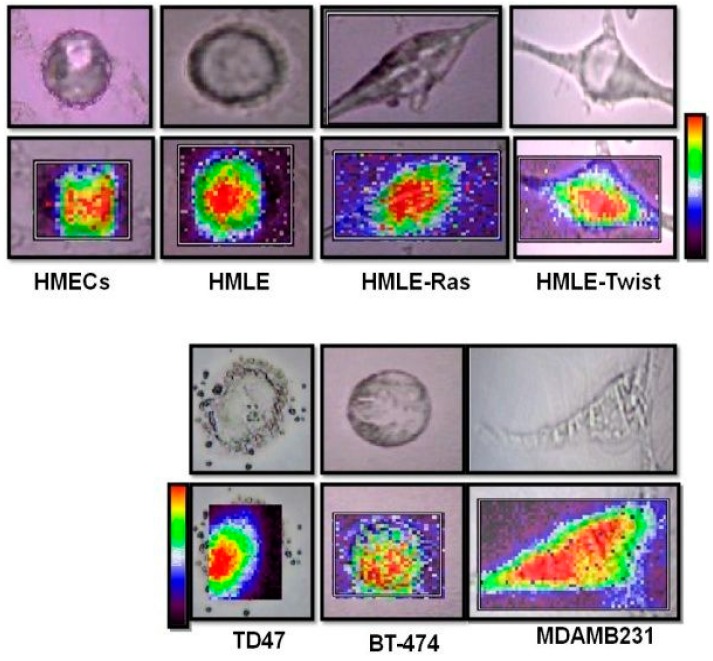
Single cell bright field images (**top**) and Raman maps created using baseline subtracted peak areas for the range 2800–3050 cm^−1^ (lipid, **bottom**) of each of the seven cell lines, HMECs, HMLE, HMLE-Ras, HMLE-Twist, T47D, BT-474, and MDAMB231. High Raman intensities are displayed in red while low Raman intensities appear in violet.

The Raman bands at 2850 cm^−1^, 2878 cm^−1^, and 2929 cm^−1^ represent CH stretching (predominantly lipid) and give information about the lipids present in the cells. We have created images using baseline subtracted peak areas for the range 2800–3050 cm^−1^ (see Figure 9). High concentrations are displayed in red, while low concentrations appear in violet. The Raman images clearly indicate that the immortalized EMT’ed HMLE-Twist cells; and the immortalized and transformed cells HMLE-Ras show an almost identical distribution of lipids throughout the cell. We see that the invasive/metastatic epithelial MDAMB231 cells show the highest distribution of lipids throughout the cells. Lower lipid levels are only evident in normal cells (HMECs) and non-invasive cells (BT-474).

## 4. Discussion

Based on the spectral differences discussed above, the biomolecular changes in invasive cell lines could be distinguished from the non-invasive cell lines. In the invasive vs. non-invasive cell comparison, we observed differences in the intensity of the bands at 1447 cm^−1^ and 1002 cm^−1^. The 1447 cm^−1^ band, which mostly corresponds to the bending of the CH_2_ group of lipids, and the 1002 cm^−1^ band, which is the phenylalanine ring breathing mode, show a prominent increase in intensity in the invasive HMLE-Twist (Figure 3A) and MDAMB231 (Figure 3B) cell lines.

At this point we can conclude that not only lipids but also proteins correlate with the transformation from non-invasive to invasive transformation. Mbaye et al. [55] found a significant increase in the amount of tryptophan and phenylalanine in cancerous tissues and concluded that these increased levels could indicate an increased risk of developing breast cancer. Our experiments also indicate a clear and intense Raman peak at 1002 cm^−1^ (phenylalanine) in the case of transformed (HMLE-Ras) cell lines, followed by invasive cell lines (HMLE-Twist and MDAMB231). Apart from phenylalanine, we have also observed that the Raman band of tryptophan at 876 cm^−1^ shows a relatively high intensity in the case of invasive cell lines.

These findings agree with the report of Nieva et al., who directly correlated lipid phenotype to high aggressiveness and metastatic spread using Raman spectroscopy [24]. They monitored the lipid phenotype associated with breast cancer malignancy by acquiring spectra from fixed breast cancer cells in the HWN window. Enhanced lipogenesis has long been known to be linked to many types of cancers e.g., prostate and breast cancer [56,57,58].

Our results are also in good agreement with previous reports that suggested [24,59,60] that lipid-rich tumors are considerably aggressive. Lipid richness was correlated with aggressiveness [61] and poor prognosis of the cancer. It was reported that lipids have a special role in controlling cell adhesion and migration [62]. These reports have been supported by clinical data, showing that lipid-rich breast carcinomas were responsible for half of the deaths of cancer patients. Intracellular lipid accumulation has also been observed at a clinical level in many types of cancers including breast cancer, with reports of n6 polyunsaturated fatty acids playing a major role in mammary tumor development [63]. Cancer progression has been associated with de novo production of fatty acids in tumor cells, which is required for the increased membrane formation during rapid cell proliferation [64]. This could possibly explain the relative intensity of the increase in the Raman bands corresponding to lipids observed in our study. It is also reported that the activation of de novo lipogenesis in metastatic cells is mainly mediated by multiple lipogenic enzymes [65,66,67]. Santana et al. [25] have reported that the high concentration of lipids in metastatic cells is because of de novo lipogenesis [26,65]. In this work, by comparing single point spectra of live cells and also staining experiments, we observed that the Raman bands related to lipids clearly showed a relatively higher intensity in all the invasive cell lines and might be considered as a useful biomarker for detecting invasive breast cancer cells.

## 5. Conclusions

Our results indicate that Raman microspectroscopy could be a promising tool for distinguishing invasive cells from non-invasive cells based on their lipid profiles. In principle, one could also quantify the lipid levels based on the relative intensities of corresponding Raman bands and a set of well-defined calibration standards, but that is beyond the scope of this work. Our results indicate that the relative enhancement of lipids could be associated with the invasiveness and aggressiveness of the cancer. It will be interesting to know the specific nature of these lipids and their associated functions. Using Raman microspectroscopy, we successfully monitored the cancer progression phases and observed that lipids could play a major role in the transformation of normal cells to invasive cells. Further confirmation was obtained with the help of Nile Red and BODIPY staining techniques. We observed significantly higher intensities of phospholipids in the invasive and transformed cell lines relative to other cell lines. The assessment of the Raman spectra acquired from each group of cells via PC-LDA analysis showed the formation of distinct clusters of normal, immortalized, transformed and invasive cells, with an overall positive classification accuracy. The subsequent evaluation of the PC-LDA model via the LOOCV approach demonstrated the potential of this analysis to correctly identify cells of different phases of breast cancer with overall convincing accuracies. Overall, the results presented in this study demonstrate that the changes observed in the Raman spectra and Raman images are sensitive indicators for the spatial distribution and relative levels of biomolecules within the cells at a single cell resolution. Therefore, this approach has the potential to be used for studying breast cancer invasiveness and tumorigenesis at the cellular level, and may facilitate the identification and functional analysis of prognostic biomarkers for breast cancer. This approach could possibly help to distinguish invasive cells from non-invasive and immortalized cells and could thus help in early diagnosis.

## Figures and Tables

**Table 1:** Raman spectral assignments.

Raman Shift (cm^−1^)	Molecular Assignment [44,45,46]
756	Tryptophan
782	cytosine and thymine
810	C05–O–P–O–C′3 phosphodiester bands
825	tyrosine (p)
851	ring breathing of tyrosine
876	Tryptophan
936	skeletal mode of polysaccharides
986	C–C or C–O in ribose
1002	ring breathing of phenylalanine
1031	C–H in phenylalanine
1065	C–N, C–C stretch (p)
1081	C–N (p), CC (l)
1095	O–P–O symmetric stretching
1125	C–N str in protein or C–O str in carbohydrates
1155, 1176	Carotenoids
1209	stretching mode in phenylalanine and tyrosine
1254	amide III β sheet
1268	amide III (α helix of protein)
1302	CH_2_ deformation of phospholipids
1318, 1339	CH_2_ twist and bend (nucleic acids, proteins, lipids)
1447	C–H def. nucleic acids, proteins, lipids
1582	adenine, guanine (nucleic acid)
1606, 1665	aromatic amino acids (p)
1654	amide I (p)
2850	CH_2_symstr (l)
2878	CH_2_asymstr (l)
2929	CH_3_symstr (p,l)
2968	CH_3_asymstr (p,l)

Abbreviations: (p) protein, (l) lipid, (sym) symmetric, (asym) asymmetric, (def) deformation, (str) stretch.

## Supplementary Materials

Electronic Supplementary Information (ESI) http://www.mdpi.com/2079-6374/6/4/57/s1: Figure S1: Phase-contrast microscopic images of the seven cell types, Figure S2: Live-dead analysis, cells were stained Trypan Blue and observed under the microscope after Raman Measurements, Figure S3: Raman spectra of the background (PBS + MgF_2_ cover-slip), Figure S4: Loading plots for the multivariate analysis of the Raman spectra (lower wavenumber range), Figure S5: Illustration of the variability of the Raman spectral intensity from highest to lowest for the lower wavenumber from live cells, Figure S6: Comparison of the intensity of the Raman band (A) 1447 cm^−1^ and (B) 2929 cm^−1^ with the BODIPY and Nile Red staining intensities, Table S1: Sensitivity and Specificity of the PC-LDA Model for the Test Set, Table S2: Sensitivity and Specificity of the PC-LDA Model for the Test Set (Identification) leave-one-out cross-validation analysis.
