# Supplementary material for: Different Phases of Breast Cancer Cells: Raman Study of Immortalized, Transformed, and Invasive Cells
Source: Biosensors (Basel). 2016 Nov 28;6(4):57. doi: 10.3390/bios6040057 (PMC5192377; doi:10.3390/bios6040057)
Supplement: Supplementary file 1 [file biosensors-06-00057-s001.pdf]

Supplementary Materials: Different Phases of Breast Cancer Cells: Raman Study of Immortalized, Transformed, and Invasive Cells

Deepika Chaturvedi, Sai A. Balaji, Vinay Kumar Bn, Freek Ariese, Siva Umapathy and Annapoorni Rangarajan

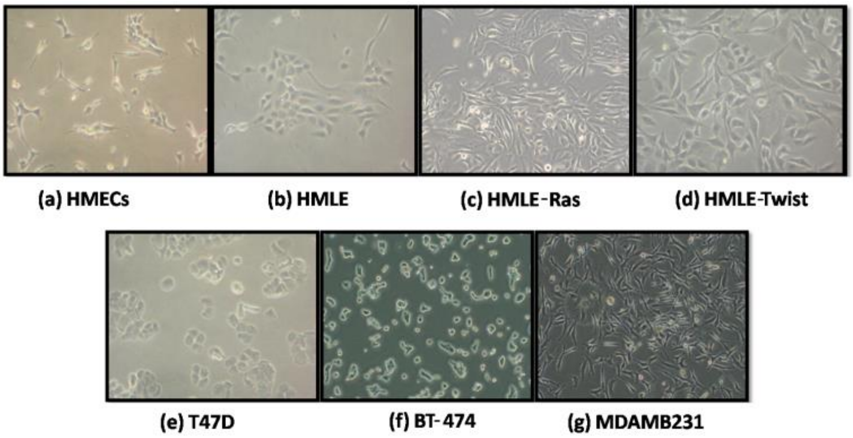

Figure S1. Phase-contrast microscopic images of the seven cell types: (**a**) HMECs; (**b**) HMLE; (**c**) HMLE-Ras; (**d**) HMLE-Twist; (**e**) T47D; (**f**) BT-474; (**g**) MDAMB231.

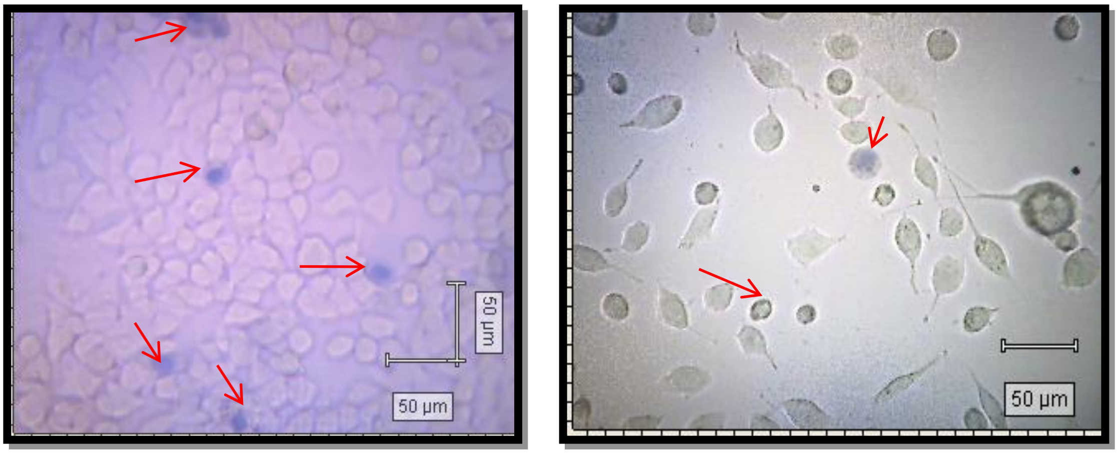

Figure S2. Live/dead analysis; cells were stained with 0.2 µg/mL, and observed under the microscope after Raman measurements, shown here for T-47D (**left**) and HMLE Ras (**right**). The majority (above 95%) of the cells were negative for Trypan lue stain; only a few cells were found dead (red arrows).

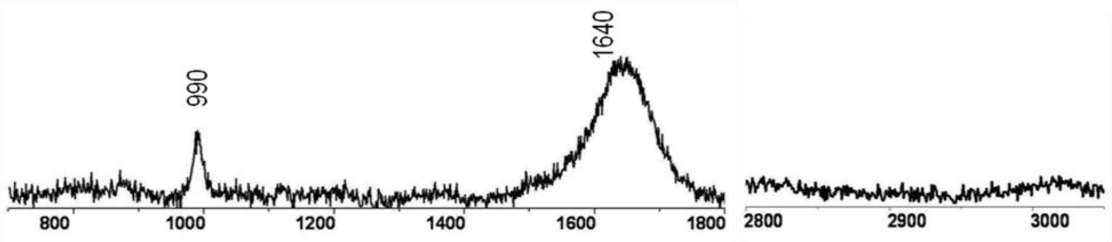

Figure S3. Raman spectra (700–1800 $cm^{-1}$and 2800–3050 cm^{-1}) of the background (PBS + MgF_2 cover-slip), acquired in 25 s and with the baseline subtracted manually.

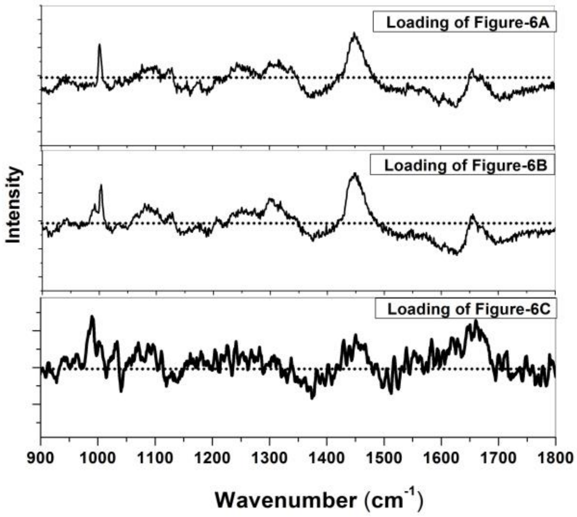

Figure S4. Loading plots for the multivariate analysis of the Raman spectra (lower wavenumber range). **Top:** HMECs, HMLE and HMLE-Ras cells (Figure 6A); **middle:** T47D, HMLE, and HMLE-Twist cells (Figure 6B); **bottom:** BT-474 and MDAMB231 cells (Figure 6C).

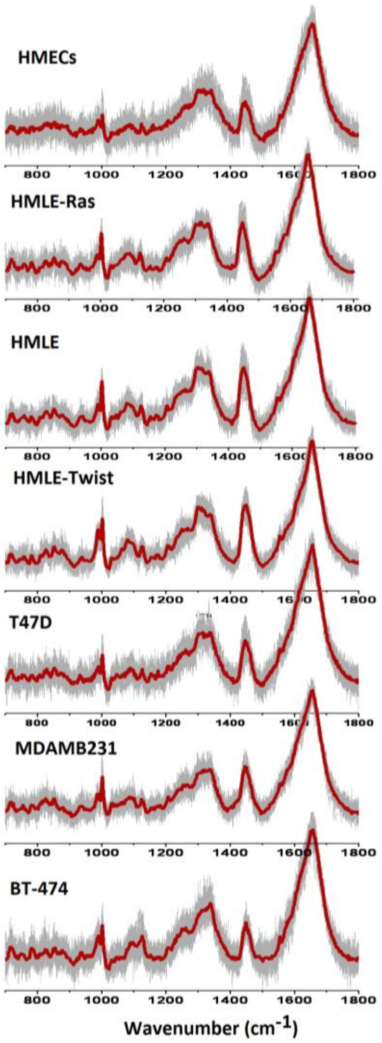

Figure S5. Illustration of the variability of the Raman spectra for the lower wavenumber range from live cells. Each spectrum (red solid line) is an average of 30 spectra. Raman spectra were acquired in 25 s and had the baseline subtracted manually. Individual spectra, from highest to lowest intensity, are depicted as gray-shaded areas.

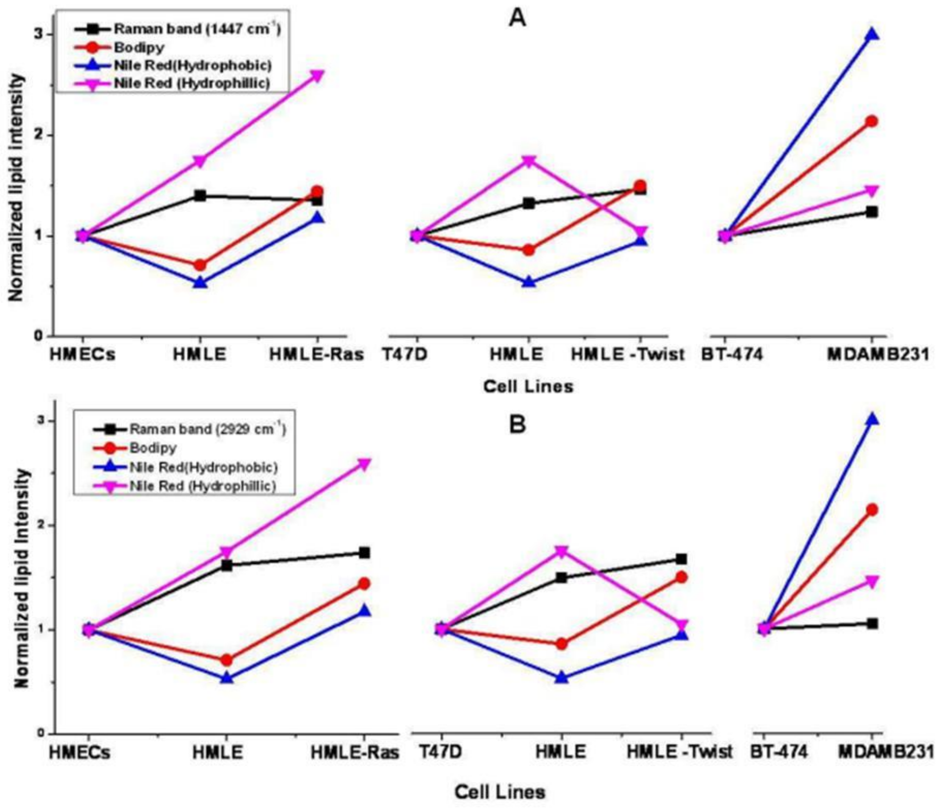

Figure S6. Comparison of the intensity of two Raman bands, (**A**) 1447 cm^{-1} and (**B**) 2929 cm^{-1}, with the BODIPY and Nile Red staining intensities. Within each group of cell lines, we have normalized the intensities to that of the control group (least invasive cell line).

Table S1. Sensitivity and specificity of the PC-LDA model for the test set.

Cell	Sensitivity (%)	Specificity (%)
normal, immortalized, and transformed cellsfor three-class separation		
HMECs	79	91
HMLE	73	81
HMLE-Ras	78	93
Accuracy	83%	
non-invasive and invasive cellsfor three-class separation		
T47D	83	92
HMLE	88	90
HMLE- Twist	79	94
Accuracy	76%	
non-invasive and invasive cellsfor two-class separation		
BT-474	100	100
MDAMB231	100	100
Accuracy	100%	

Table S2. Sensitivity and specificity of the PC-LDA model for the test set (identification) leave-one-out cross-validation analysis (LOOCV).

**Cell**	**Sensitivity (%)**	**Specificity (%)**
normal, immortalized, and transformed cellsfor three-class separation		
HMECs	100	95
HMLE	88	94
HMLE-Ras	88	98
Accuracy	92%	
non-invasive and invasive cellsfor three-class separation		
T47D	91	94
HMLE	88	94
HMLE- Twist	80	89
Accuracy	86%	
non-invasive and invasive cellsfor two-class separation		
BT-474	100	100
MDAMB231	100	100
Accuracy	100%	
